# Supplementary material for: Genome-Wide Identification and Characterization of the NF-YA Gene Family and Its Expression in Response to Different Nitrogen Forms in Populus × canescens
Source: Int J Mol Sci. 2022 Sep 23;23(19):11217. doi: 10.3390/ijms231911217 (PMC9570100; doi:10.3390/ijms231911217)
Supplement: Supplementary file 1 [file ijms-23-11217-s001.zip › 20220818 figure.pdf]

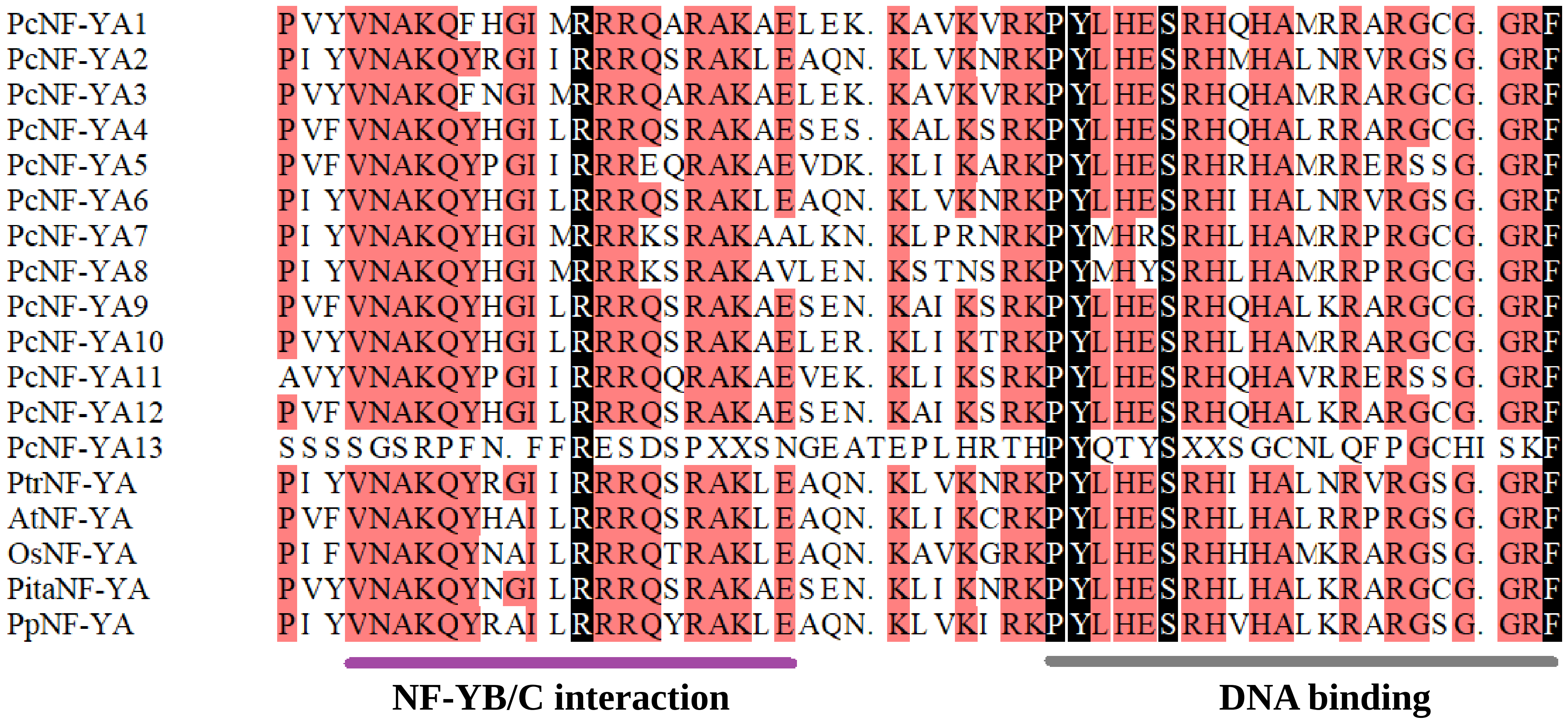


**Figure S1. Multiple alignments of *P. × canescens* PcNF-YA family members**. The gray line indicates the CBF_NF-YA conserved domain. The purple line indicates the NF-YB/C interaction.

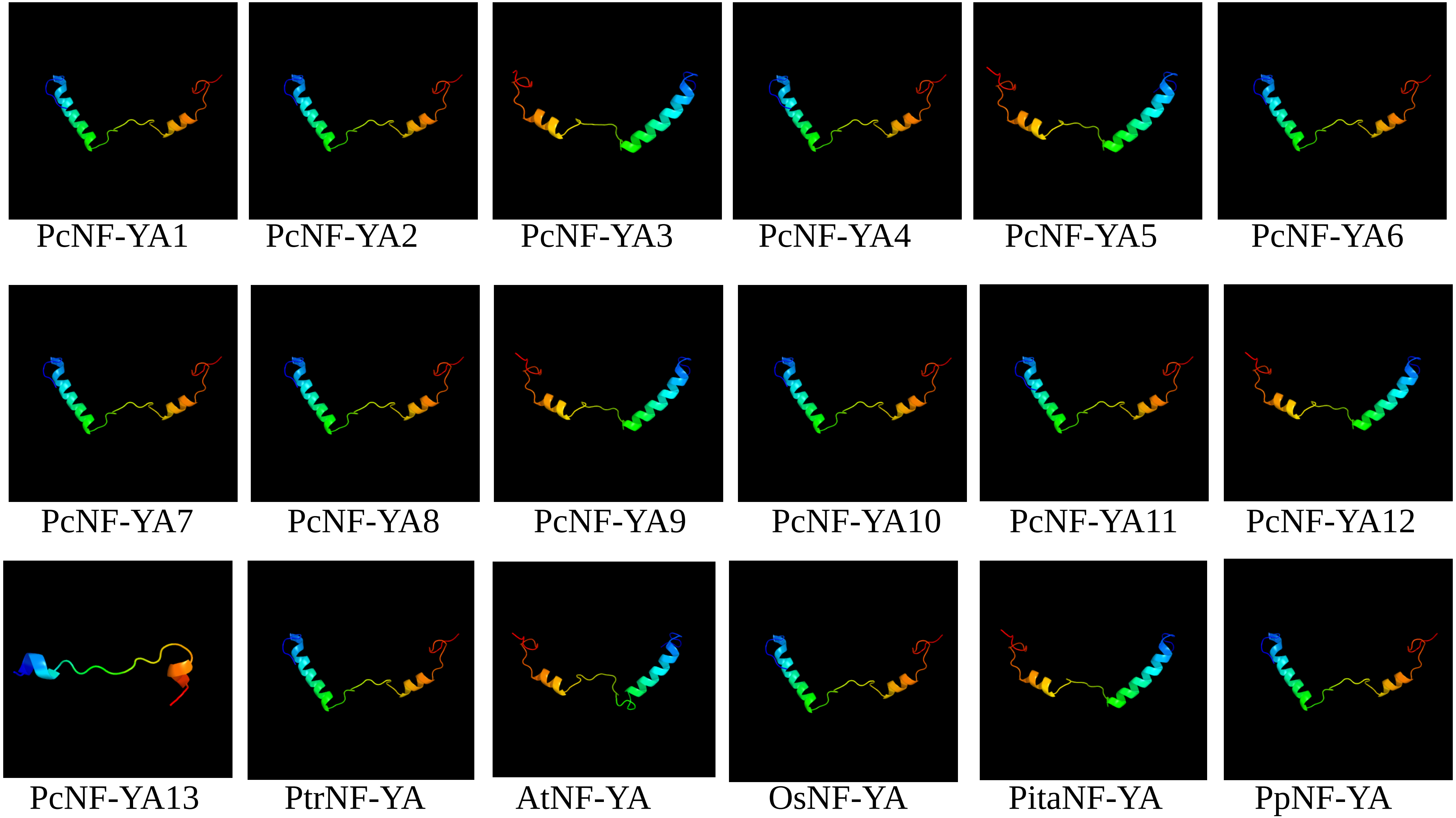


**Figure S2. Predicted tertiary structure of 13 PcNF-YA proteins in *P. × canescens*.** The tertiary structures are colored in rainbow order and represent the N to C termin.

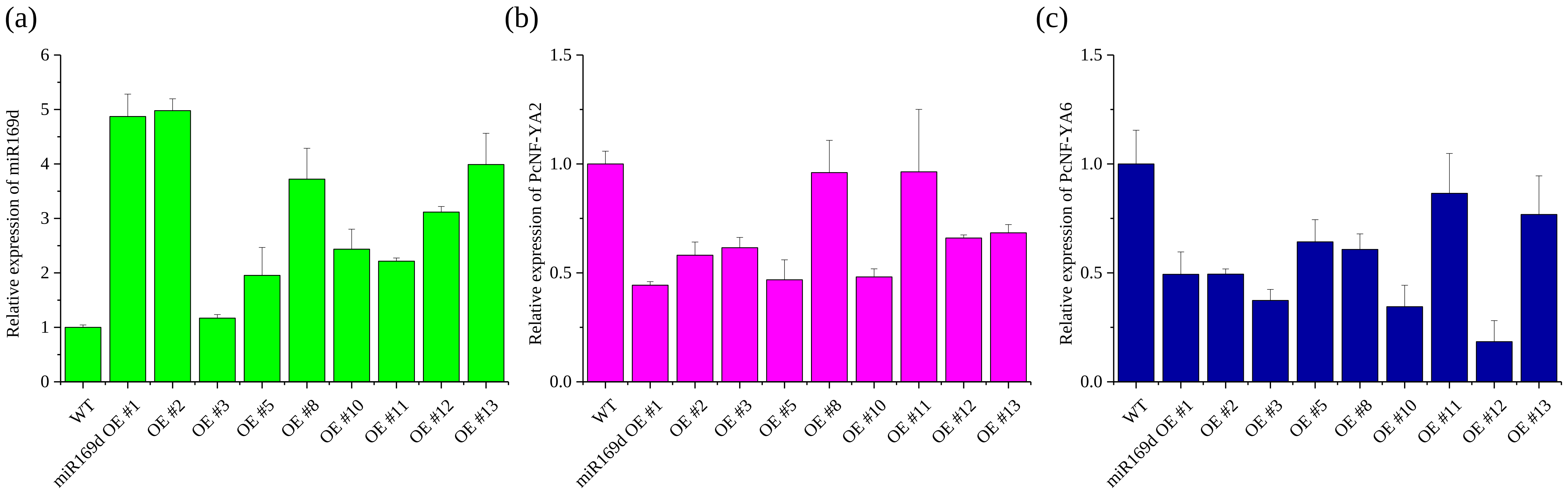


**Figure S3. Expression analysis of the miR169d (a) and *PcNF-YA2/6* genes (b, c) with RT-qPCRs in 9 transgenic *Populus* lines.** The data are expressed as the mean±SD (n=3).
